# The Structural, Thermal and Morphological Characterization of Polylactic Acid/Β-Tricalcium Phosphate (PLA/Β-TCP) Composites upon Immersion in SBF: A Comprehensive Analysis

**DOI:** 10.3390/polym16050719

**Published:** 2024-03-06

**Authors:** Sondes Ftiti, Sandra C. Cifuentes, Awatef Guidara, Joaquín Rams, Hassib Tounsi, Juan P. Fernández-Blázquez

**Affiliations:** 1 Laboratory of Advanced Materials (LR01ES26), National Engineering School of Sfax, University of Sfax, Sfax 3038, Tunisia; sondes.ftiti@enis.tn (S.F.); awatef.guidara@enis.tn (A.G.); hassib.tounsi@fss.usf.tn (H.T.); 2 Department of Applied Mathematics, Materials Science and Engineering and Electronic Technology, Universidad Rey Juan Carlos (URJC), 28933 Móstoles, Spain; joaquin.rams@urjc.es; 3 IMDEA Materials Institute, C/Eric Kandel 2, 28906 Getafe, Spain

**Keywords:** polylactic acid, β-tricalcium phosphate, gel permeation chromatography, crystallinity, solvent casting technique

## Abstract

Biocomposite films based on PLA reinforced with different β-TCP contents (10%, 20%, and 25%wt.) were fabricated via solvent casting and immersed in SBF for 7, 14, and 21 days. The bioactivity, morphological, and thermal behavior of composites with immersion were studied using scanning electron microscopy (SEM), energy-dispersive X-ray (EDX) microanalysis, weight loss (W_L_), X-ray diffraction (XRD), Fourier transform infrared spectroscopy (FTIR), differential scanning calorimetry (DSC), thermogravimetric analysis (TGA), and gel permeation chromatography (GPC). This broad analysis leads to a deeper understanding of the evolution of the polymer–filler interaction with the degradation of the biocomposites. The results showed that β-TCP gradually evolved into carbonated hydroxyapatite as the immersion time increased. This evolution affected the interaction of β-TCP with PLA. PLA and β-TCP interactions differed from PLA and carbonated hydroxyapatite interactions. It was observed that β-TCP inhibited PLA hydrolysis but accelerated the thermal degradation of the polymer. β-TCP retarded the cold crystallization of PLA and hindered its crystallinity. However, after immersion in SBF, particles accelerated the cold crystallization of PLA. Therefore, considering the evolution of β-TCP with immersion in SBF is crucial for an accurate analysis of the biocomposites’ degradation. These findings enhance the comprehension of the degradation mechanism in PLA/β-TCP, which is valuable for predicting the degradation performance of PLA/β-TCP in medical applications.

## 1. Introduction

Polylactic acid (PLA) is a thermoplastic polymer derived from various natural resources such as corn starch, sugarcane, biomass, and other vegetable wastes through fermentation [1]. Because PLA is biodegradable, it has a broad range of applications, particularly in the fields of orthopedics, craniofacial surgery, and bone grafts [2]. Due to their adequate physical and mechanical properties, the number of medical devices produced by PLA is increasing rapidly and is expected to rise in the near future [3]. PLA presently occupies the most important position in medical resorbable implants given its favorable characteristics, such as compatibility with human tissue, nontoxicity, durability, biodegradability, and rigidity [4,5,6]. The literature presents numerous approaches [4,7,8,9,10,11,12] to improve PLA bioactivity, resorbability, and hydrophilicity with the combination of bioactive fillers and surface engineering. One of the most employed is β-tricalcium phosphate (β-TCP), which has attracted much attention in medical research due to its excellent biocompatibility, osteoconduction ability, and reabsorption when implanted. PLA-TCP composites have already been designed using methods such as 3D printing, fused deposition modeling [13,14,15], gas foaming [16,17], and melt mixing [10,18,19]. In our study, we used a solvent casting technique, known for its cost-effectiveness and the production of highly homogeneous films. The in vitro bioactivity of this material can be evaluated by the assay developed by Kokubo et al. [20] using simulated body fluid (SBF). It is well established that bioactive materials immersed in an SBF solution engage in ion exchange processes, ultimately forming hydroxyapatite layers on the material’s surface, a common feature of their bioactivity. The bioactivity of PLA/β-TCP biocomposites has been demonstrated by various authors using different techniques such as X-ray diffraction, Fourier transform infrared spectroscopy (FTIR-ATR) analysis, scanning electron microscopy (SEM) and EDX [12,21,22,23]. Thermogravimetric analysis (TGA) has also been utilized to demonstrate that tricalcium phosphate (TCP) is transformed into hydroxyapatite (HA) [12]. Some other researchers have investigated how the molecular weight of PLA decreases with immersion time in SBF solution. However, the influence of β-TCP particles on the hydrolysis of PLA in PLA/β-TCP composites has not been addressed and remains unclear [22,23].

While studying the thermal properties of a composite in its initial state and after immersion in the physiological environment (SBF) helps to understand the changes in polymer–filler interaction with immersion time, little effort has been devoted to studying the thermal behavior of PLA/β-TCP biocomposites with immersion time in physiological media. This work represents the first comprehensive study that investigates the bioactivity, morphology, and thermal behavior of PLA/β-TCP biocomposites in combination. Understanding the impact of the filler (β-TCP) on the molecular weight, stability, and crystallization of the polymeric matrix (PLA) is crucial to comprehending the degradation behavior of these biocomposites.

The primary objective of this paper is to perform a thorough evaluation of the in vitro degradation of PLA/nβ-TCP biocomposites through the examination and correlation of their bioactivity, morphology, and thermal performance. To achieve this, samples before and after immersion in SBF for 7, 14 and 21 days were analyzed using scanning electron microscopy (SEM), energy-dispersive X-ray (EDX) microanalysis, weight loss (WL), X-ray diffraction (XRD), Fourier transform infrared spectroscopy (FTIR), differential scanning calorimetry (DSC), thermogravimetric analysis (TGA), and gel permeation chromatography (GPC). The obtained results are essential for a deeper understanding of the degradation mechanism of PLA/β-TCP films, which is valuable for predicting the performance of these resorbable biocomposites in medical applications.

## 2. Materials and Methods

### 2.1. Materials

PolyLite^TM^ polylactic acid (PLA) with a melt flow index (MFI) ranging between 7 and 11 g/10 min, density varies between 1.17 and 1.24 g/cm^3^, and a molecular weight of 200,000 g/mol (Computer Aided Technologies, CAT, Sfax, Tunisia) was used as the matrix. The reinforcement was β-tricalcium phosphate (β-TCP) with chemical formula Ca_3_(PO_4_)_2_, high purity (>96%), and average particle size (d_50_) of 10.0 μm, which was purchased from Sigma-Aldrich (St. Louis, MO, USA). The solvent used in this study was dichloromethane (DCM, purity 99.5%) with the chemical formula CH_2_Cl_2_ (Sigma-Aldrich).

### 2.2. Methods

#### 2.2.1. Fabrication of PLA/nβ-TCP Biocomposites

**Figure 1:**
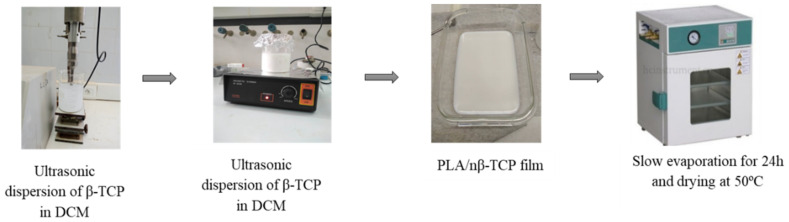
Preparation process of PLA/n β-TCP biocomposites.

For the preparation of PLA matrix composite films using the solvent casting technique, a 13% PLA solution by weight was used employing dichloromethane. Firstly, processing was optimized to find the appropriate conditions for the manufacture of the polylactide-based composite films. Different percentages of β-TCP (10, 20, and 25 wt.%) were incorporated to study their influence on PLA. In order to facilitate the homogenization process of the reinforcement in the polymeric matrix, sonication equipment was used with a 750 W ultrasonic sound amplifier. TCP sonification was performed with a 38% amplitude and a frequency range of 0.5–0.5 Hz for 2 min to achieve a high degree of dispersion and stability of the β-TCP powder particles in PLA, followed by the addition of the corresponding amount of PLA. The mixture was further stirred until complete dissolution for approximately 3 h. Afterward, the blend was poured into a rectangular crystallizer, and the solvent was left to evaporate for 24 h at room temperature. Finally, the obtained films were dried at 50 °C for 48 h under vacuum, as shown in Figure 1. Three biocomposites were prepared with different amounts of β-TCP. Samples were labeled as PLA/n β-TCP, with n corresponding to the weight percentage of β-TCP (n = 10 wt.%, 20 wt.%, and 25 wt.%).

#### 2.2.2. Simulated Body Fluid (SBF) Preparation

SBF was prepared using a thermostatically controlled bath at a temperature close to 36.5 °C. Reagents (NaCl, NaHCO_3_, KCl, K_2_HPO_4_3H_2_O, MgCl_2_6H_2_O, HCl (1 M), CaCl_2_, Na_2_SO_4_, TRIS, and HCl (1 M)) were dissolved one by one in 700 mL of demineralized water. The mixture volume was then brought to one liter with demineralized water buffered to the normal pH of human plasma (pH = 7.42) using Tris and hydrochloric acid. The nominal ion concentrations of SBF are presented in [24,25].

#### 2.2.3. In Vitro Degradation Experiment in Static SBF

An in vitro degradation test was conducted in simulated body fluid (SBF) with ion concentrations similar to human blood plasma. A constant contact surface was maintained between the sample and the physiological fluid. For the measurements to be reproducible, the ratio of sample surface area (S) to the SBF volume (V) was kept constant. We used an S/V ratio equal to 0.1 cm^−1^. Samples were immersed in 10 mL of SBF solution with an initial pH value of 7.50 ± 0.1 at a constant temperature of 37 °C. At a predetermined control period of time (7, 14, and 21 days), the samples were removed from the SBF solution, rinsed gently with deionized water, and placed in an oven programmed to a fixed temperature of 37 °C for the appropriate periods. The samples were labeled as PLA/nβ-TCP-md, with m corresponding to the duration of immersion (0, 14, and 21) in days (d).

#### 2.2.4. Material Characterization

Scanning electron microscopy (SEM) analysis was performed on the surface of PLA/nβ-TCP films using a Hitachi SEM model S-3400 (Tokyo, Japan). The films were observed under an accelerated voltage of 15 kV.

From this analysis, the topographical characteristics of particles were obtained from secondary electron signals. In addition, elemental compositions of the different biocomposites were analyzed. The samples were sputter-coated with gold and then analyzed using an EDX spectrometer (EDS Bruker Xflash 5010, Karlsruhe, Germany) to measure the Ca/P ratio.

XRD patterns were recorded with a PANalytical X’Pert PRO diffractometer (Almelo, The Netherlands) with Cu-Kα radiation (λ = 1.541 Å), and the range of diffraction angles was scanned from 5° to 80° counted in 2θ at a scan speed of 2 °/min.

Infrared spectroscopy measurements were conducted using a PerkinElmer Frontier FTIR Spectrometer (Waltham, MA, USA) for biocomposites based on PLA. In each measurement, acquisitions were carried out in the range between 400 and 4000 cm^−1^; the number of scans was 64, and the resolution was 4 cm^−1^.

PLA/nβ-TCP composites were analyzed by differential scanning calorimetry (DSC) (DSC 25, TA Instruments, New Castle, DE, USA). The specimens (4–9 mg) were subjected to heating from 20 to 200 °C at a rate of 5 °C/min under a nitrogen atmosphere. The gas flow was 50 mL/min. The thermal properties were observed from the second heating scan after cooling the sample back to 20 °C at a rate of 5 °C/min. The degree of crystallinity (*χ_c_*) for samples was determined according to the following Equation (1) [26]:

(1) $$\chi_{c}=\frac{\Delta H_{m}-\Delta H_{cc}}{w\times \Delta H_{m}^{0}}\times 100\%$$

where ∆H_m_ and ∆H_cc_ are the enthalpies of melting and cold crystallization, respectively; w and ∆H^0^_m_ are the weight fraction of PLA and the melting enthalpy of 100% crystalline PLA, respectively. ∆H^0^_m_ is the enthalpy of melting for 100% crystalline PLA (∆H^0^_m_ = 93.7 J/g) [27].

The thermal stabilities of PLA and PLA/nβ-TCP biocomposites before and after biodegradation experiments were investigated using thermal analysis with a device manufactured by TA Instruments (TGA Q50 thermogravimetric analyzer). The samples (10–17 mg) were heated at 20–900 °C with a N_2_ flow rate of 10 mL/min and a heating rate of 10 °C/min. The obtained thermograms were analyzed using Universal Analysis 2000 software developed by TA Instruments.

The molecular weight (M_W_) distribution of the PLA/nβ-TCP composites was measured by gel permeation chromatography (GPC) (GPC 2414, Waters, Milford, MA, USA). The system used a Waters 2424 refractive index detector and a series of narrow polystyrene standards, with tetrahydrofuran as the mobile phase.

To measure the percentage of water uptake (W_uptake_), the samples were removed from the SBF solution and weighed with an electronic balance to obtain the corresponding wet mass (m_wet_) and then dried to obtain the mass of the dried sample (m_r_). The water uptake percentage was calculated using Equation (2) [28]:

(2) $$W_{uptake}(\%)=\frac{m_{wet}-m_{r}}{m_{r}}\times 100$$

The percentage of the weight loss (W_L_) was obtained from the weight difference between the initial mass (m_0_) and the mass of the dried sample (m_r_), calculated according to Equation (3) [29,30]:

(3) $$W_{L}(\%)=\frac{m_{0}-m_{r}}{m_{0}}\times 100$$

The pH values of the immersing SBF solutions were measured with a pH meter (pH −200 L) at predetermined time intervals under a physiological condition (37 °C ± 1.5 °C).

In this study, the concentrations of calcium and phosphorous ions present in the solution after immersion were analyzed using atomic absorption spectroscopy (AAS) and UV–Vis spectroscopy, respectively. AAS was employed to quantify the concentration of calcium ions by measuring the absorption of light by free atoms in a gaseous state. A signal processor integrated changes in the absorbed wavelength, presenting them as energy absorption peaks on the red output at distinct wavelengths. The atomic absorption spectrometer (ICE 3000 series, Illkrich, France) was employed to detect the concentration of calcium ions within each solution of the immersed films. As the solution was drawn into a flame, the sample’s element underwent transformation into atomic vapor. In the ground state, atoms absorbed specific radiation emitted by the hollow cathode lamp of calcium. The wavelength of this emitted radiation from the lamp is directly proportional to that absorbed by the atoms in the flame.

Simultaneously, UV–Vis Spectroscopy was utilized to determine the concentration of phosphorous ions. We used the spectrophotometric method by measuring the absorbance of the blue complex formed from phosphorus ions with ammonium molybdates and ascorbic acid at a wavelength of 880 nm. These analytical techniques provided valuable insights into the calcium and phosphorous content in the solution following immersion, enabling a comprehensive understanding of the changes occurring during the immersion process. The results are shown as the mean ± standard deviation of three samples for each soaking time in SBF.

## 3. Results

### 3.1. Morphological Changes and Ca/P Ratio Evolution

**Figure 2:**
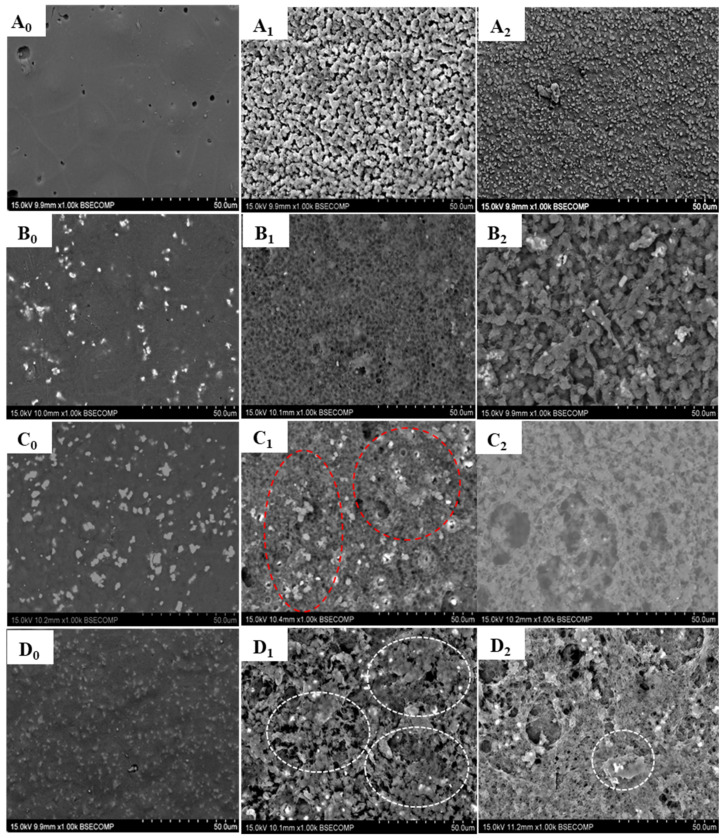
SEM micrographs of the surfaces of PLA (**A_0_**–**A_2_**) and PLA/nβ-TCP composites as a function of the β-TCP content: 10 wt.% (**B_0_**–**B_2_**), 20 wt.% (**C_0_**–**C_2_**), and 25 wt.% (**D_0_**–**D_2_**) before and after incubation in SBF.

Figure 2 shows the SEM micrographs of neat PLA, PLA/10β-TCP, PLA/20β-TCP, and PLA/25β-TCP before soaking in SBF (Figure 2(A_0_–D_0_)) and after immersion in the SBF solution for 14 days (Figure 2(A_1_–D_1_)) and 21 days (Figure 2(A_2_–D_2_)). The selection of images at specific time lapses was based on choosing the most representative results and samples that exhibited significant differences in morphology. After the immersion of PLA for 14 and 21 days, the surface became porous, as shown in Figure 2(A_1_,A_2_). In addition, Figure 2(B_1_–D_1_) indicate that the surface of composites after immersion in the SBF for 14 days is porous with the presence of small pores; the amount of particles of β-TCP decreases, and particles with different shapes and sizes are observed (red circles on the pictures). Other particles are disbanded (white circles). However, after 21 days, the surface of PLA/nβ-TCP composites (Figure 2(B_2_–D_2_)) entirely changed as the TCP particles were further dissolved; in addition, the surface had very high porosity and larger open pores than the composites after 14 days. This porosity is of great significance for the application of scaffolds in bone tissue engineering, and it is needed to guide the regeneration of bone tissue and promote the growth of new bone [31,32]. Table 1 presents the changes in the Ca/P ratios of the composites before and after incubation for 14 and 21 days using SBF. The Ca/P molar ratios increased with immersion time for all the composites. Before soaking in SBF, the Ca/P molar ratio was about 1.5, which then increased to 1.651 after incubation for 21 days. The representative EDX spectra complementary to Table 1 are provided in Supplementary Materials.

### 3.2. Structural Analysis

**Figure 3:**
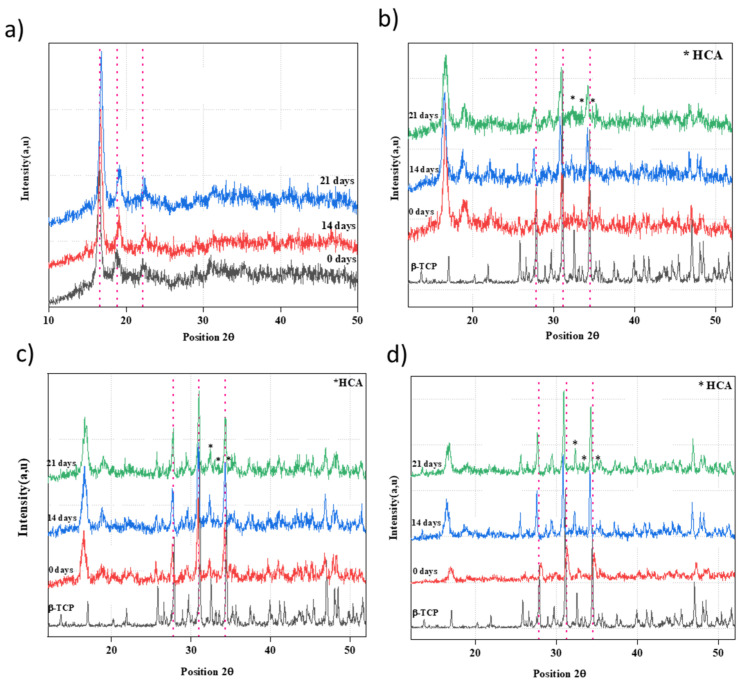
XRD profiles of PLA and PLA/nβ-TCP biocomposites before and after immersion in SBF solution: (**a**) PLA; (**b**) PLA/10β-TCP; (**c**) PLA/20β-TCP; (**d**) PLA/25β-TCP. (red dot lines refer to the main peaks of PLA (**a**) and β-TCP (**b**–**d**)).

Figure 3 illustrates the XRD profiles of PLA/nβ-TCP biocomposites at different immersion times. The XRD profiles for all materials show that they all present a PLA matrix with high crystallinity. From the XRD profile of PLA (Figure 3a), one can see three distinct diffraction peaks located at 16.9°, 19.3°, and 22.5°, corresponding to the (110/200), (203), and (210) planes, respectively, of the PLA α form [33]. With an increase in immersion time, the PLA profile reveals a shift in peak positions to a higher 2θ value. Furthermore, according to PLA/nβ-TCP profiles, there is a shift in β-TCP main peak positions, tending toward a lower 2θ value. As seen in the PLA/25β-TCP profile, the peaks shift from 28.09° to 26.67°, 31.04° to 30.88°, and 34.75° to 34.21° as the duration of immersion increases from 0 days to 21 days (Figure 3d). Such a shift in the PLA/nβ-TCP peak position can be attributed to the formation of a new compound on the surface. This can be clearly seen from Figure 3b–d. PLA immersion in SBF did not lead to carbonated hydroxyapatite (HCA) formation. It is well established that PLA alone is not able to induce the formation of calcium phosphates on its surface, as reported in the literature [8]. However, carbonated hydroxyapatite peaks were detected at 32.36°, 33.53°, and 34.36° for PLA/n β-TCP biocomposite surfaces (Figure 3b–d) almost overlapping the other TCP peaks.

### 3.3. Fourier Transform Infrared Spectroscopy (FTIR)

**Figure 4:**
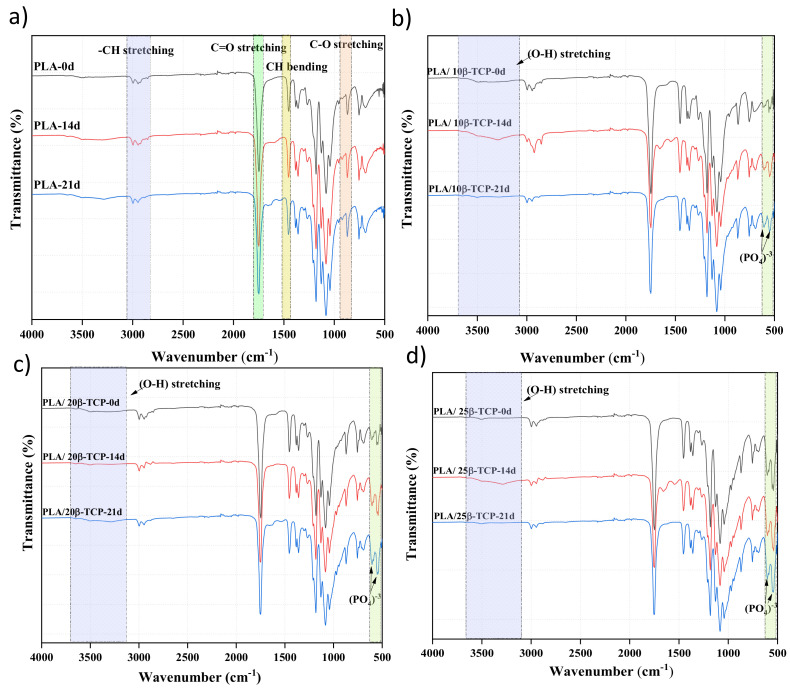
FTIR spectra of PLA and PLA/nβ −TCP biocomposites with immersion time: (**a**) PLA; (**b**) PLA/10β −TCP; (**c**) PLA/20β −TCP; (**d**) PLA/25β −TCP.

Figure 4 displays the FTIR spectra of PLA and PLA/nβ-TCP biocomposites at various immersion times. PLA exhibits peaks at 2993 and 2853 cm^−1^, which are the typical stretching bands of symmetric and asymmetric vibrations of the C-H bond of the CH_3_ groups, and at 1750 and 1080 cm^−1^ for C=O and C-O-C stretching vibration peaks, respectively. The band at 1450 cm^−1^ is attributed to the C-H stretching vibration in methyl groups, while the bands at 1381 and 1362 cm^−1^ are attributed to symmetric and asymmetric vibrations of -CH- bending. Additionally, the peak at 752 cm^−1^ is attributed to the C-C stretching vibration [21,32]. On the other hand, β-TCP shows typical absorption bands at 1088, 550, and 608 cm^−1^ (bending out of the plane of the PO_4_^3−^ group); 1037 cm^−1^ (the asymmetric vibration of the PO_4_^3−^ group); and 962 cm^−1^ (the asymmetric vibration of the PO_4_^3−^ group) [12]. Furthermore, bands at approximately 1392 cm^−1^ (ν_3_) and 863 cm^−1^ (ν_2_) are attributed to CO_3_^2−^, and they overlap with peaks in PLA. The spectra also exhibit an absorption band at 3440.99 cm^−1^ corresponding to the hydroxyl stretching vibration (O-H). In addition, immersion in SBF and the appearance of new compounds on the surface of materials can affect the exposed chemical groups and their intensities [34]. As shown in Figure 4, the intensity of PO_4_^3−^ group in PLA/nβ-TCP composites increases with the β-TCP content and immersion time, which can be attributed to the growth of apatite layers [35].

### 3.4. Evolution of pH, Calcium and Phosphorous Ions in SBF, and Weight Loss in Composites

**Figure 5:**
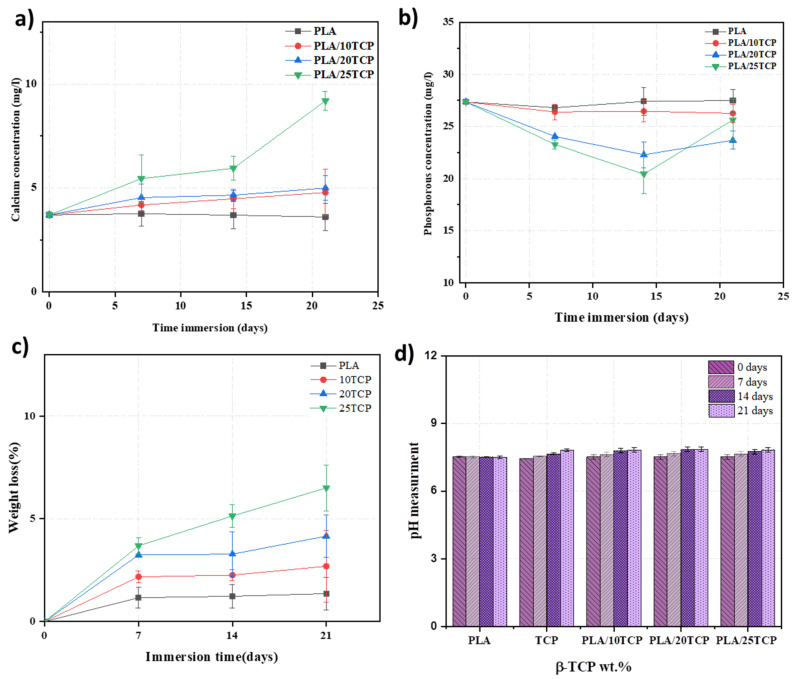
Evolution of calcium (**a**) and phosphorous concentrations (**b**) during the immersion of PLA/nβ-TCP biocomposites in SBF solution; (**c**) percentage of mass losses after 21 days of immersing PLA and PLA/nβ-TCP biocomposites in SBF; (**d**) evolution of pH with immersion time.

The calcium and phosphorous concentrations in SBF solutions of PLA and PLA/nβ-TCP biocomposites were measured after 7, 14, and 21 days (Figure 5a,b). Figure 5c presents the evolution of weight loss for all materials with immersion time. The variation in the pH values of the SBF solution is illustrated in Figure 5d. The calcium concentration in the initial SBF solution was about 3.73 mg/L. It increased sharply during the first 7 days in all composites and remained constant or continued to increase at a lower rate for PLA/10β-TCP and PLA/20β-TCP. However, in PLA/25β-TCP, it continued to increase until it reached 8.96 mg/L on the 21st day.

On the other hand, the phosphorous concentration in the initial SBF solution was about 27.34 mg/L, which slightly decreased or remained almost constant for PLA/10β-TCP. Nevertheless, for the other biocomposites, phosphorus concentration decreased rapidly until the 14th day and then increased. Calcium and phosphorus concentrations for PLA remained constant during the immersion test. As shown in Figure 5c, the pure PLA underwent much less degradation than the composites, with a weight loss of 1.4% after soaking in the SBF for 21 days. At the end of the immersion period, the weight losses of PLA/10β-TCP, PLA/20β-TCP, and PLA/25β-TCP increased to 2.7%, 4.1%, and 6.5%, respectively.

In fact, during the immersion test, the biocomposites released Ca^2+^ ions, resulting in a loss of mass [36,37]. Consequently, the calcium concentration in SBF increased. After 7 days, PLA/10β-TCP and PLA/20β-TCP biocomposites exhibited almost constant mass loss, corresponding to a stable calcium concentration as well (Figure 5a). In contrast, PLA/25βTCP continued to release calcium into the SBF solution due to the dissolution of β-TCP in SBF [9,30]. Figure 5d shows a slight increase in pH values during the immersion time for β-TCP and PLA/β-TCP composites. This increase can be related to the variations in sample composition and the ion release rate. For instance, the release of Ca^2+^ from biocomposites into SBF accelerates H_2_O ionization and increases OH^−^ concentration, leading to an increment in pH [38].

### 3.5. Water Uptake of PLA and PLA/nβ-TCP with Immersion Time

**Figure 6:**
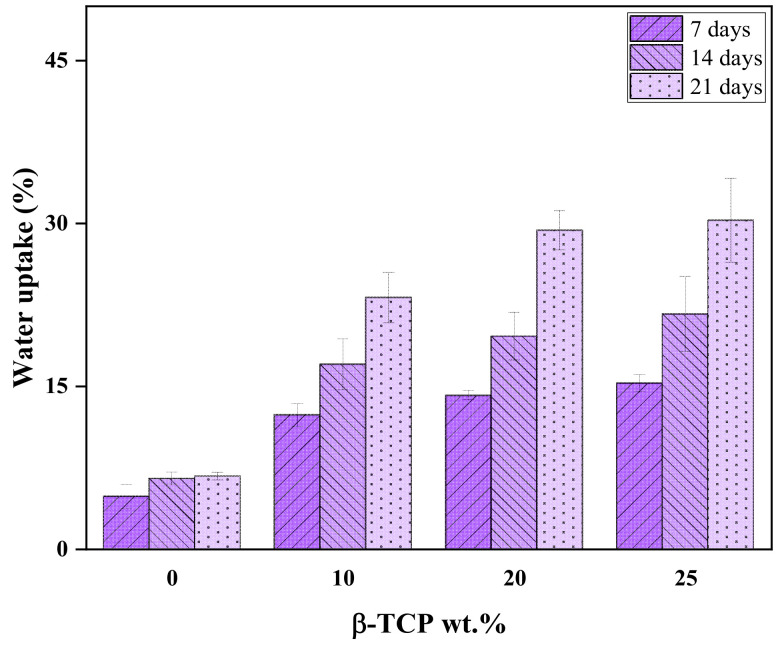
Percentage of water uptake after immersion in SBF of PLA and PLA/nβ-TCP biocomposites.

Figure 6 illustrates the water uptake of PLA and PLA/nβ-TCP biocomposites at different periods of time. As shown in Figure 6, the neat PLA had a slow water absorption rate after 14 and 21 days of immersion, whereas the water absorption of PLA/nβ-TCP biocomposites increased with the incorporation of β-TCP fillers of 10%, 20%, and 25%, and as the immersion time in the SBF solution increased. This can be explained by the strong hydrophobicity of PLA due to its hydrophobic ester bond (-COOR) and side methyl (-CH_3_), which tends to repel water [39] and result in the best hydrophilic performance of β-TCP, which enhances the water–filler interaction [40].

### 3.6. Effect of β-TCP and Immersion Time on the Molecular Weight of PLA

**Figure 7:**
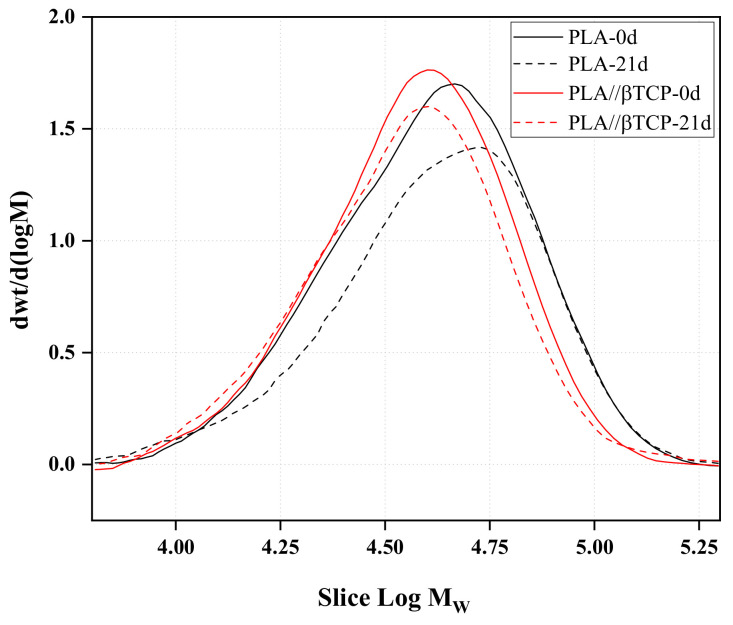
The molecular weight distribution of PLA and PLA/25β-TCP.

The molecular weight distributions of PLA and PLA/25β-TCP are presented in Figure 7. The number average molecular weight (M_n_), the weight average molecular weight (M_w_), and the polydispersity index (PDI) before and after incubation in SBF solution for 21 days are listed in Table 2. The molecular weight of the neat PLA film significantly decreased after immersion in SBF. However, the molecular weight (M_w_) of PLA/25β-TCP films was slightly lower than their initial value. Additionally, the polydispersity index (PDI), which measures the breadth of the polymer’s molecular weight distribution (MWD), increased with degradation time. This implies that the incorporation of TCP particles within PLA hinders PLA hydrolysis.

### 3.7. Effect of β-TCP and SBF Solution on the Thermal Degradation of PLA

**Figure 8:**
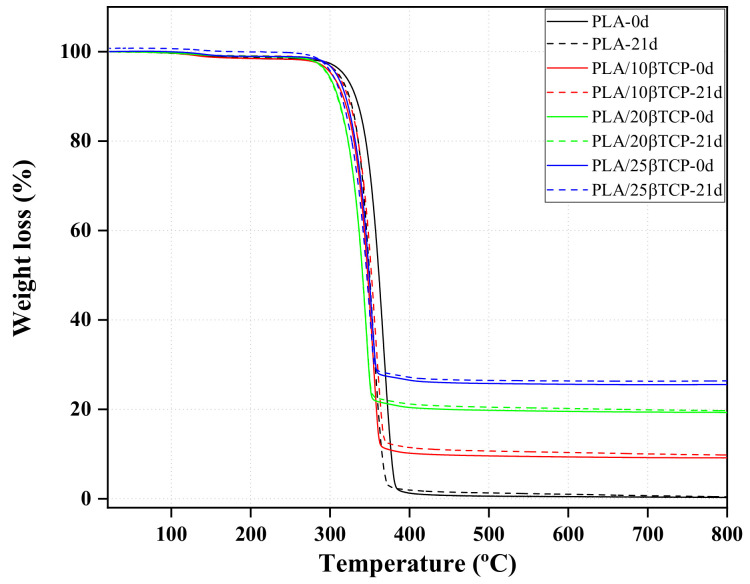
Thermogravimetric analysis of PLA and PLA/nβ-TCP biocomposites before and after immersion in SBF for 21 days.

**Figure 9:**
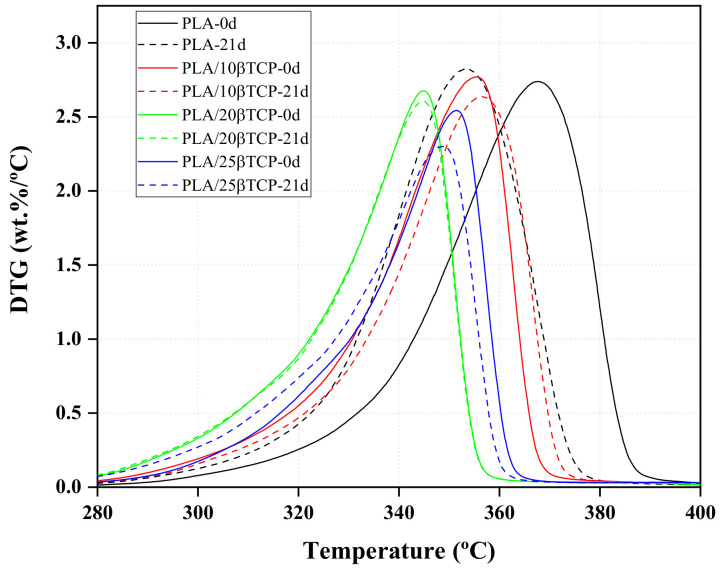
DTG curves of PLA and PLA/nβ-TCP before and after immersion in SBF for 21 days.

Thermogravimetric analysis (TGA) and the first derivative of weight loss (DTG) of PLA and PLA/nβ-TCP nanocomposites before and after immersion for 21 days in SBF are shown in Figure 8 and Figure 9, respectively. The aim was to analyze the changes in the thermogravimetric behavior and residual mass at both day 0 and after 21 days. The TGA curves show that the decomposition of all samples can be divided into two stages. The first weight loss between 50 and 200 °C is attributed to physisorbed water [32]. The second main weight loss between 300 and 390 °C is related to the degradation of PLA. The degradation process of PLA and PLA/nβ-TCP biocomposites after immersion for 21 days in the SBF solution can be explained by the corresponding characteristic temperatures obtained from TGA and the degradation temperature (T_d_) acquired from DTG (Table 3). Comparing nonimmersed samples (materials at 0 days), we found that materials with β-TCP degraded at lower temperatures, indicating that these particles accelerated the thermal degradation of PLA. After immersion in SBF solution for 21 days, PLA exhibited a decrement in T_d_ from 345.12 to 338.17 °C (around 7 °C). However, for PLA/25β-TCP, the decrement in T_d_ was only 3 °C (from 328.10 to 325.93 °C); for PLA/10β-TCP and PLA/20β-TCP, T_d_ remained stable. As is well known, thermal stability is related to changes in the molecular weight of materials [41,42]. In fact, as shown in Figure 9, the decrease in the degradation temperature (T_d_) of PLA and PLA/25β-TCP during immersion time can be attributed to changes in the molecular weight distributions after immersion in the SBF solution. Additionally, according to the GPC results, since the molecular weight of the neat PLA decreased rapidly compared to that of PLA/25β-TCP, lower minimum stability was observed in PLA. Also, the residual mass of PLA, PLA/10β-TCP, PLA/20β-TCP, and PLA/25β-TCP were 0.19%, 9.06%, 19.24%, and 25.58%, respectively. These findings indicate that our technique allows for the fabrication of homogeneous films containing different percentages of β-TCP. On the other hand, the remaining weights of PLA/10β-TCP, PLA/20β-TCP, and PLA/25β-TCP slightly increased after immersion in SBF for 21 days. For example, it changed from 9.06% to 9.51% in PLA/10β-TCP and from 19.24% to 19.52% in PLA/20β-TCP.

### 3.8. Influence of β-TCP and Immersion Time on the Thermal Behavior of PLA

**Figure 10:**
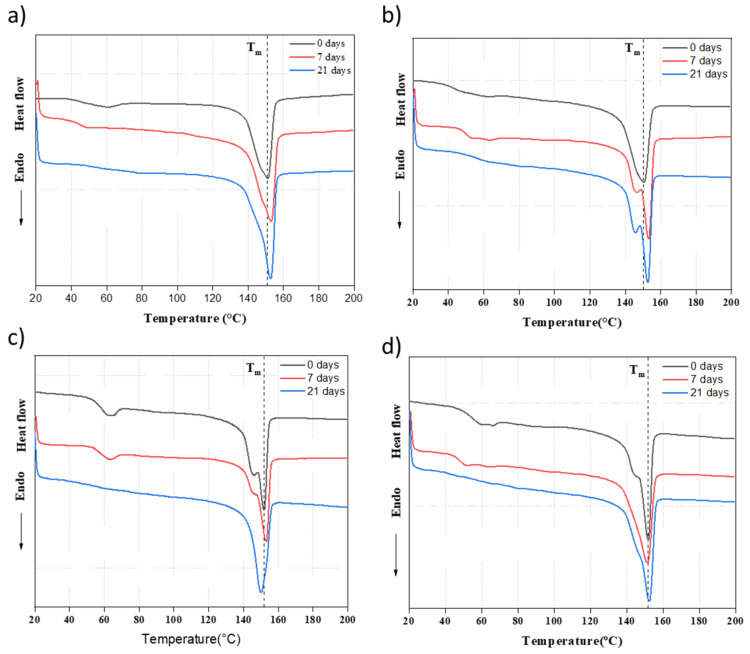
DSC curves during the first heating cycle of PLA/β-TCP films at a scanning rate of 5 °C/min: (**a**) PLA; (**b**) PLA/10β-TCP; (**c**) PLA/20β-TCP; (**d**)PLA/25β-TCP.

The DSC curves of PLA and PLA/nβ-TCP during the first heating show the thermal history of biocomposites, as illustrated in Figure 10. The degrees of crystallinity (χ_c_) of all samples before and after soaking them in SBF for 21 days are presented in Table 4. The DSC curves provide evidence indicating that the solvent cast materials have high crystallinity given the absence of a cold crystallization peak. The crystallinity of the materials ranged from 23.9% in PLA/25β-TCP and 29.5% in PLA. The crystallinity of the composites decreased with the increment in β-TCP at 0 days. PLA crystallinity increased from 29.5% to 34.7% when this material was immersed in SBF for 21 days. However, the crystallinity of PLA/25β-TCP increased from 23.9% to 24.4% when immersed in SBF for 21 days.

**Figure 11:**
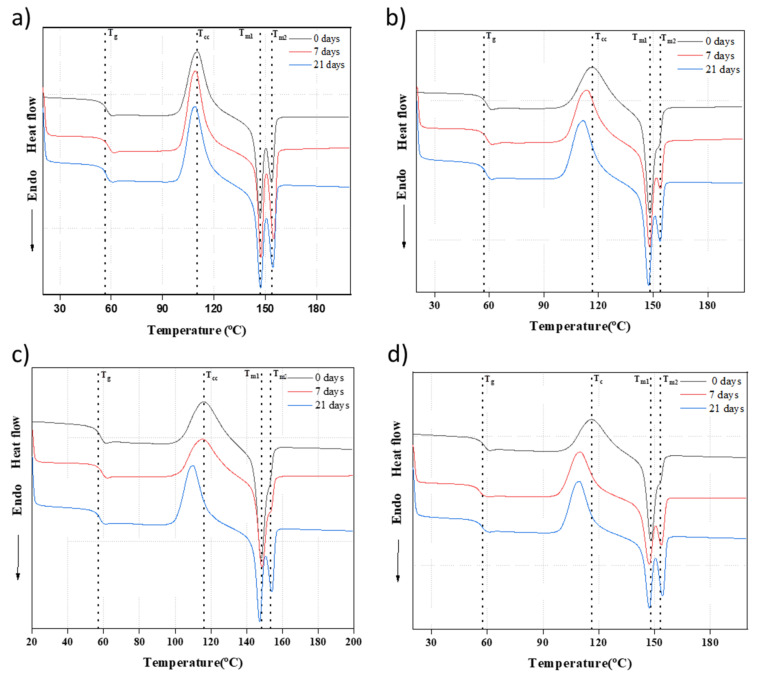
DSC curves during the second heating cycle of PLA/β-TCP films at a scanning rate of 5 °C/min. Glass transition (T*_g_*), cold crystallization (*T_cc_*), and melting temperature (*T_m_*) are marked with black dashed lines: (**a**) PLA; (**b**) PLA/10β-TCP; (**c**) PLA/20β-TCP; (**d**) PLA/25β-TCP.

**Figure 12:**
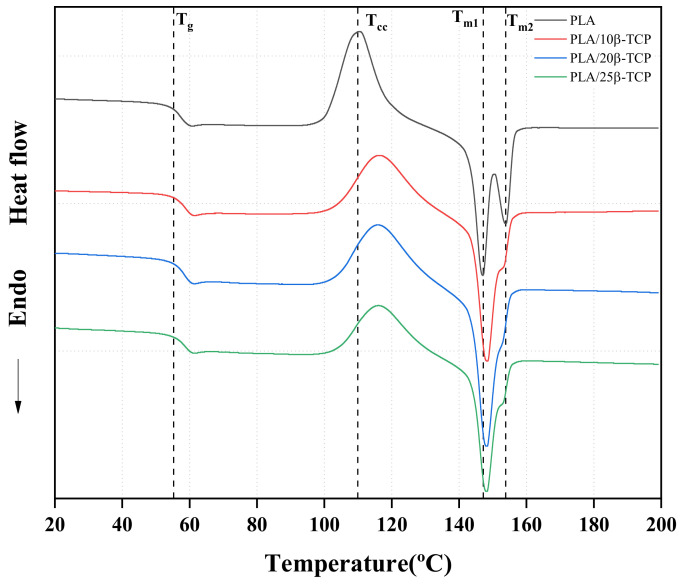
DSC curves during the second heating cycle of PLA and PLA/nβ-TCP films before immersion in SBF.

Figure 11 displays the DSC profiles during the second heating of the neat PLA and PLA/nβ-TCP biocomposites with immersion time (0, 7, and 21 days). All materials initially underwent a glass transition and then exhibited an exothermic peak due to cold crystallization and finally an endothermic double peak. Table 4 presents the characteristic temperatures (glass transition T_g_, cold crystallization T_cc_, and melting temperatures T_m1_ and T_m2_) for PLA and PLA/nβ-TCP biocomposites before and after immersion in the SBF solution for 21 days. Regarding the glass transition temperature, with the addition of β-TCP and immersion time, we found no evidence of a change in Tg, as shown in Figure 11 and Table 4. This implies that the filler did not affect the mobility of polymeric chains or the molecular weight reduction after 7 and 21 days in SBF. When comparing all the materials at 0 days, as depicted in Figure 12, β-TCP influenced the cold crystallization temperature as the T_cc_ peak in composite materials was observed at higher temperatures than in PLA. For PLA, T_cc_ did not change with immersion time. However, for the composites, the T_cc_ peak was detected at lower temperatures when the material was immersed in SBF. Therefore, β-TCP retarded the cold crystallization of PLA, but the compound resulting from the exchange of ions between β-TCP and SBF had a better interaction with PLA than β-TCP. The compound allowed for the acceleration of cold crystallization in the polymer. In the composites immersed for 7 and 21 days in SBF, crystals were formed at lower T_cc_ and then melted at lower T_m1_, as shown in Table 4. The double melting peak is due to the melting–recrystallization–melting phenomenon [43,44].

## 4. Discussion

PLA and calcium phosphates, especially β-TCP, have wide applications in reconstructive bone surgery and dentistry [45]. The current research involves the analysis of the degradation of biocomposites based on PLA/β-TCP in SBF. Various percentages of β-TCP powders were incorporated into PLA, and its bioactivity, morphological, structural, and thermal properties were investigated as a function of immersion time in simulated body fluid (SBF). The aim of immersing materials in an SBF solution was to simulate the physiological conditions of the human body to assess the reactivity of materials and study the effect and nucleation of β-TCP on the polymeric matrix. This method makes it possible to analyze the ability of materials to promote the formation of an apatite layer, similar to the hydroxyapatite present in human bones, and thus assess their potential for biomedical applications such as bone implants.

The EDX analysis revealed a Ca/P ratio value of 1.65, which closely aligns with the stoichiometric value of CHA reported for the composite materials after 21 days of immersion in the SBF solution. In this sense, via the EDX analysis of PLA/TCP, Redondo et al. [12] pointed to the presence of apatitic calcium phosphate with Ca/P ratios greater than 1.50, approximating the stoichiometric ratio of hydroxyapatite (HA) after soaking the samples in SBF for 14 days. Ben Rjeb et al. [46] reported on the evolution of the Ca/P ratio with immersion time in SBF for tricalcium phosphate–magnesium oxide composites, attributing it to the deposition of the apatite layer.

In our studies, we observed that, for PLA/β-TCP composites, the evolution of calcium and phosphorus concentrations in SBF depended on the content of β-TCP within PLA. The calcium concentration consistently increased for all the composites, with PLA/25β-TCP inducing the highest increment in Ca^2+^ in the SBF solution. The calcium release profile was consistent with weight loss, directly proportional to the amount of β-TCP in the composite. Regarding the phosphorus concentration, it remained constant or slightly decreased for PLA/10β-TCP during the immersion time, while for higher β-TCP contents, the phosphorus concentration initially decreased and then increased after 14 days.

The release of calcium (Ca) and phosphorus (P) ions of the PLA/β-TCP composites exhibit differences when compared with calcium phosphate ceramics (β-TCP, HA, and biphasic calcium phosphate). It is well known that two processes occur simultaneously when a calcium phosphate ceramic is exposed to a physiological environment: dissolution and precipitation. If the dissolution rate exceeds the rate of precipitation or deposition, there is an increment in Ca and P ions in the SBF solution [47,48,49,50]. Conversely, when the dissolution rate is lower than the precipitation rate, there is a decrement in Ca and P ions in the SBF solution [51,52]. In our study, the increase in Ca and the decrease in P indicate that the dissolution of β-TCP and apatite precipitation occurred simultaneously. Regarding other studies on polymer/β-TCP composites, Park et al. [14] reported an increase in Ca^2+^ in SBF. However, they did not report the evolution of P in their physiological media. We found that pH increased with time for β-TCP and PLA/β-TCP. pH changes in β-TCP and PLA/β-TCP biocomposites are related to the variations in sample composition and the ion release rate. For instance, the release of Ca^2+^ from biocomposites into SBF accelerates H_2_O ionization and increases OH− concentration, leading to an increment in pH [44]. Additionally, several studies reported the formation of apatite on phosphate ceramics with an increment in pH [32,38,39].

In the present study, using XRD and FTIR and by measuring the Ca/P ratio, we confirmed that, over time, the β-TCP particles dissolved, evolved, and changed into carbonated hydroxyapatite (HCA) on the surface of the biocomposites. This result agrees with the findings of Backes et al. [7], who studied the immersion of the same type of composites in an SBF solution and detected HCA formation after incubation for 21 days. Studies from Kang et al. [21] have revealed the formation of octacalcium phosphate (OCP) and hydroxyapatite (HAp) on the surface of PLLA/TCP scaffolds after incubation in static SBF and dynamic SBF for 28 days.

The observed increase in phosphate peak intensity in FTIR with immersion time is evidence of the progressive formation of apatite, as indicated by the evolution of the vibrational signatures characteristic of apatite crystallization [34,35,53].

The identified peaks of carbonated hydroxyapatite (HCA) are consistent with those reported in the literature [54,55].

According to thermogravimetric analysis, the remaining weight increased in all composites with immersion time. This increment has been considered an indication of apatite formation in the literature [12,56]. In our study, we observed that β-TCP accelerated the thermal degradation of PLA. The onset degradation temperature of PLA decreased with increasing TCP percentage and immersion time (Table 3). Similar results were obtained by Ferri et al. [57], who demonstrated that PLA reinforced with β-TCP underwent a decrease in the onset degradation temperatures with an increment in the β-TCP content. Ferri et al. [57] also studied the effect of HA on the thermal degradation of PLA and found that, in contrast to what occurred with β-TCP, HA improved the thermal stability of PLA. Similarly, Bauer et al. [9] recently reported that HA particles increased the activation energy and initial decomposition temperature of PLA. This implies that β-TCP and HA exert different effects on the polymeric matrix’s thermal degradation.

Nevertheless, after immersing the biocomposites in SBF, particles within the polymeric matrix accelerated the cold crystallization of PLA. This implies that the apatite formed from the ion exchange within β-TCP and SBF had a better interaction with PLA than β-TCP. As β-TCP particles evolved over time, the matrix–filler interaction evolved as well. This finding reveals a new perspective on the study of PLA/β-TCP degradation. It is therefore important to consider the evolution that β-TCP particles undergo with immersion in SBF for an accurate analysis of biocomposite degradation.

Concerning the effect of β-TCP on the evolution of the molecular weight of PLA, our results of GPC clearly indicate a slight change in the molecular weight of PLA containing 25% of β-TCP particles with immersion time, compared with a higher decrement in the pure PLA’s molecular weight. PLA degrades over time in the SBF solution due to hydrolysis, which breaks down the polymer chains into smaller molecules [14,23]. Our results demonstrate that β-TCP particles retarded the hydrolysis of PLA. Rakmae et al. [58] found a similar result on the effect of HA particles on the molecular weight of PLA. They indicated that the molecular weight of PLA/HA composites decreased slower than the molecular weight of the neat PLA because the buffer solution could barely penetrate into the inner sides of the composites due to the good interface between the PLA and the HA particles. They suggested that the hydrolysis of the PLA matrix was hindered by less water availability within the polymeric chains. Thus, the SBF solution needed more time to penetrate the PLA composites and hydrolyze them. However, they did not perform water uptake studies and did not prove their hypothesis on the effect of HA particles hindering the diffusion of water within the composite. In our research, we performed studies on the water uptake of PLA/TCP composites, and our results led to a different conclusion from that of Rakmae et al. [58].

**Figure 13:**
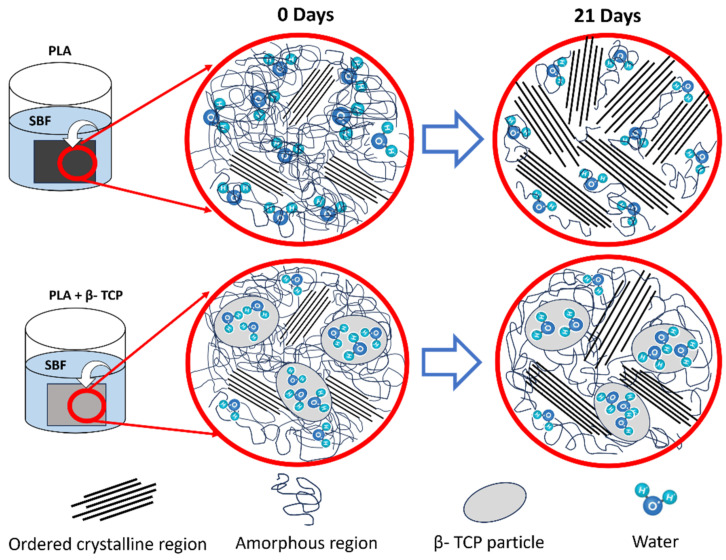
Schematic illustrating the proposed degradation mechanism of neat PLA and PLA with β-TCP particles.

Our studies on water uptake show that the β-TCP particles in PLA enhanced the uptake of the SBF solution. Water uptake was directly proportional to the amount of filler within the composites. So, why does the presence of β-TCP hinder PLA hydrolysis instead of promoting it? From the joint analysis of water uptake, DSC, GPC, and TGA results, we can conclude that water remains in the particles, which are highly porous and hydrophilic, and to a lesser extent in polymeric molecules. Figure 13 presents a schematic showing the proposed degradation mechanism of both the neat PLA and PLA with β-TCP.

It is well known that the presence of water/fluids facilitates the mobility of chains, leading to the organization of a semicrystalline polymer into more structured crystals. Chor et al. [59], who studied the in vitro degradation of poly(lactic-co-glycolic acid) (PLGA) with immersion in simulated body fluid (SBF), concluded that immersion in this fluid induced the crystallization of PLGA. When PLA is exposed to water, hydrolysis occurs as water molecules interact with the ester linkages in PLA, breaking them down. This process leads to a reduction in the molecular weight of PLA. Simultaneously, the presence of water enables the mobility of chains, fostering the formation of new crystalline regions and contributing to an overall increase in crystallinity (Figure 13).

Our DSC results showed that the increment in crystallinity after immersion time for the neat PLA was higher than that for the biocomposites, and this rise did not significantly alter the melting temperature. However, the width of the peak increased, primarily due to the appearance of a second endothermic peak, or a shoulder, at a lower temperature. This peak corresponds to the melting of new crystals formed at the immersion temperature (37 °C). These crystals were smaller and less perfect, which is why their melting occurred at a lower temperature.

As the β-TCP filler content increased in the composite, the increment in crystallinity of biocomposites was smaller with immersion time. The increment in crystallinity after immersion points to the greater mobility of the polymeric chains, which allows the chains to organize in crystals. We can conclude that the mobility of the polymeric chains is reduced by the presence of β-TCP, as the increment in crystallinity decreases with the β-TCP content. We confirm that the mobility of PLA chains within composites is hindered by β-TCP presence, and we can also state that the hydrolysis of PLA is hindered due to the low availability of water. In the case of PLA/β-TCP, water molecules may become trapped within the structure of β-TCP particles. This entrapment of water within the ceramic component diminishes its availability for direct interaction with the polymeric chains of PLA, resulting in a decreased rate of hydrolysis. Consequently, there is a less pronounced reduction in molecular weight. Similarly, with less water available, there is reduced chain mobility, resulting in a smaller increase in crystallinity compared to pure PLA (Figure 13). These results are essential for a deeper understanding of the degradation mechanism of PLA/β-TCP films, providing valuable insights for predicting the performance of these resorbable biocomposites in medical applications.

## 5. Conclusions

PLA biocomposites containing 10, 20, and 25 wt.% β-TCP were successfully fabricated via solvent casting technique. In the present study, using XRD and FTIR and by measuring the Ca/P ratio, we confirmed that, over time, the β-TCP particles dissolved, evolved, and changed into carbonated hydroxyapatite (HCA) on the surface of the biocomposites. The increase observed in FTIR regarding the phosphate peak intensity throughout immersion time provides conclusive evidence of the progressive formation of apatite. Regarding TGA analysis, the percentage of residual mass provides evidence of the good dispersion of β-TCP in the PLA matrix, and the increment in this percentage can be due to the formation of apatite in the composites after immersion in the SBF solution. The increase in Ca and the decrease in P indicate that the dissolution of β-TCP and apatite precipitation occurred simultaneously, which is indicative of changes in the Ca and P concentrations in the media. The mass loss was directly proportional to the amount of β-TCP in the composite. β-TCP particles accelerated the thermal degradation of PLA. Additionally, it was found that β-TCP retarded the cold crystallization of PLA, whereas the compound resulting from the exchange of ions between β-TCP and SBF accelerated the cold crystallization of PLA. This implies a better matrix–filler interaction between PLA and the ceramic filler after immersion in SBF than between the polymeric matrix and β-TCP. It is therefore important to consider the evolution that β-TCP particles undergo with immersion in SBF for an accurate analysis of the biocomposites’ degradation.

β-TCP particles contribute to the inhibition of PLA hydrolysis. This phenomenon can be attributed to water retention in ceramic particles, which possess a high hydrophilic characteristic. As a result, the hydrolysis of the PLA matrix is hindered due to the reduced availability of water in the polymeric chains. In DSC analysis, we found that the increment in the crystallinity of PLA was more significant than that in composites with β-TCP given the limited availability of water in the polymeric chains. These results are essential for a deeper understanding of the degradation mechanism of PLA/β-TCP films, providing valuable insights for predicting the performance of these resorbable biocomposites in medical applications. PLA/β-TCP composites have potential applications in orthopedics, including the development of bone scaffolds and implants. In the form of films, these composites can be used in dentistry, particularly as periodontal barrier membranes. Further in vitro and in vivo experiments are necessary to fully demonstrate the potential of these materials for orthopedic and dental applications.

## Figures and Tables

**Table 1:** Evolution of Ca/P ratio of PLA/nβ-TCP composites after 14 and 21 days of immersion in SBF.

Samples	Ca/P Ratio		
	0 Day	14 Days	21 Days
PLA/10βTCP	1.502 ± 0.061	1.565 ± 0.075	1.621 ± 0.130
PLA/20βTCP	1.506 ± 0.065	1.570 ± 0.011	1.641 ± 0.058
PLA/25βTCP	1.500 ± 0.011	1.574 ± 0.069	1.651± 0.036

**Table 2:** Average molecular weight data for PLA and PLA/25β-TCP derivatives, as determined by gel permeation chromatography (GPC).

Samples	M_w_ (kDa)	M_n_ (kDa)	PDI (M_w_/M_n_)
PLA-0d	47.168	35.162	1.341
PLA-21d	36.027	22.650	1.590
PLA/25β-TCP-0d	56.368	42.622	1.322
PLA/25β-TCP-21d	55.782	38.917	1.433

**Table 3:** Thermal degradation results of PLA and PLA/nβ-TCP samples.

Biocomposites	* T_onset_ (°C)	Mass Change (%)	Residual Mass (%)	** T_d_ (°C)
PLA-0d	345.12	97.46	0.19	368.00
PLA-21d	338.17	96.85	0.13	353.47
PLA/10β-TCP-0d	339.41	88.34	9.06	355.84
PLA/10β-TCP-21d	335.00	87.63	9.51	357.20
PLA/20β-TCP-0d	332.43	78.39	19.24	345.21
PLA/20β-TCP-21d	330.37	77.97	19.52	345.21
PLA/25β-TCP-0d	328.10	72.53	25.58	351.60
PLA/25β-TCP-21d	325.93	72.90	25.68	348.93

* T_onset_: initial temperature of the degradation curve, ** T_d_: degradation temperature.

**Table 4:** Thermal transition temperatures and their associated enthalpies for PLA and PLA/βTCP biocomposites before and after immersion in SBF solution for 21 days.

Samples	T_g_ (°C)	T_cc_ (°C)	T_m1_ (°C)	T_m2_ (°C)	χ_c_ (%)
PLA-0d	56.8	110.1	147.0	153.7	29.5
PLA-21d	56.0	109.1	147.3	154.5	34.7
PLA/10βTCP-0d	56.2	116.5	148.4	153.8	27.3
PLA/10βTCP-21d	56.9	111.4	147.5	153.8	29.1
PLA/20βTCP-0d	57.7	116.0	148.1	154.0	23.0
PLA/20βTCP-21d	56.7	109.5	147.3	154.0	27.5
PLA/25βTCP-0d	57.4	116.1	148.1	153.1	23.9
PLA/25βTCP-21d	56.0	109.4	147.2	153.3	24.4

T_m_ and T_cc_ ± 0.5 °C, T_g_ ± 2 °C, Xc ± 4%.

## Data Availability

The data presented in this study are available upon request from the corresponding author.

## Supplementary Materials

The following supporting information can be downloaded at: https://www.mdpi.com/article/10.3390/polym16050719/s1.
