# Supplementary material for: The Structural, Thermal and Morphological Characterization of Polylactic Acid/Β-Tricalcium Phosphate (PLA/Β-TCP) Composites upon Immersion in SBF: A Comprehensive Analysis
Source: Polymers (Basel). 2024 Mar 6;16(5):719. doi: 10.3390/polym16050719 (PMC10934208; doi:10.3390/polym16050719)
Supplement: Supplementary file 1 [file polymers-16-00719-s001.zip › polymers-2884149-supplementary.pdf]

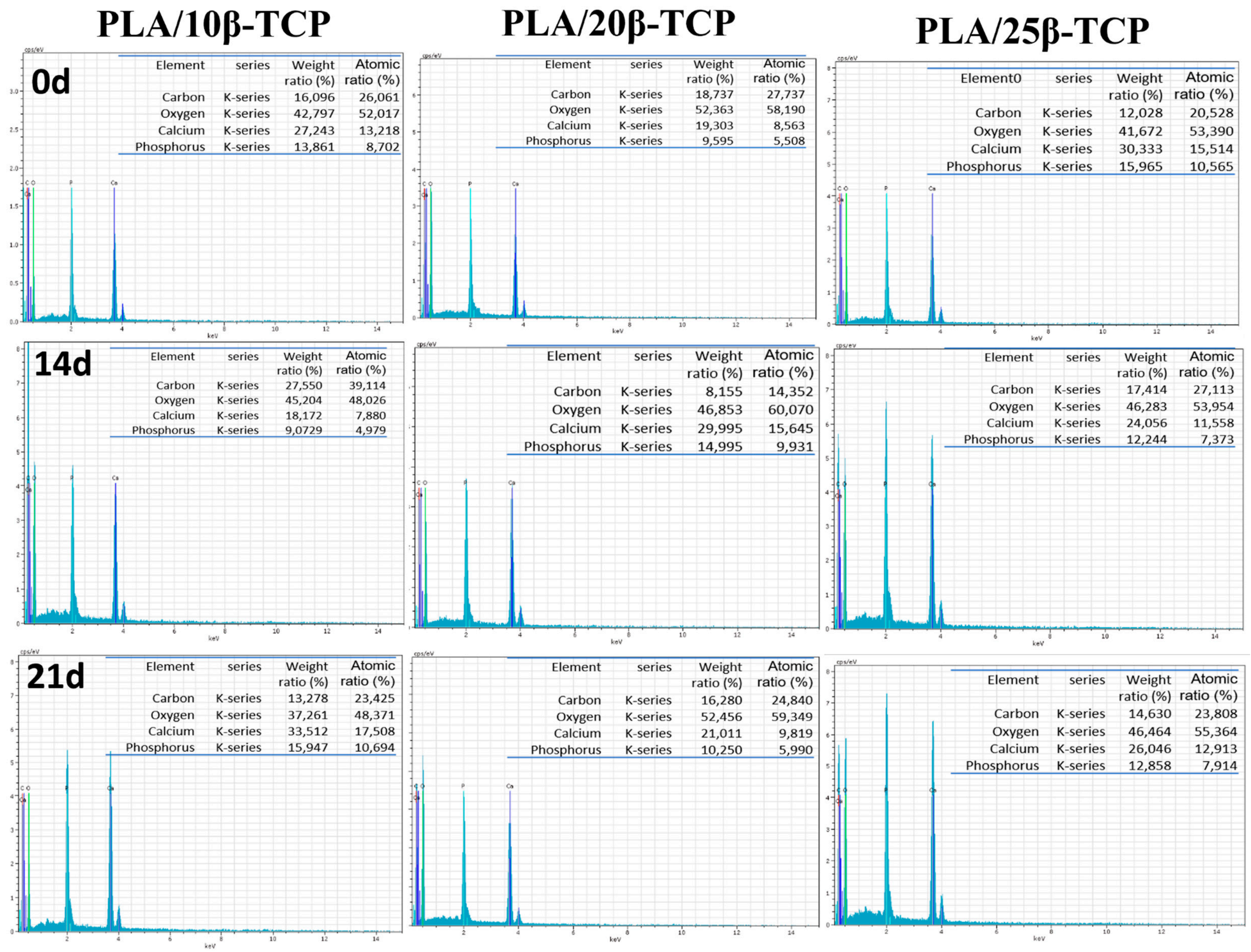

Supplementary Figure: Representative EDX spectra complementary to Table 1 Evolution of Ca/P ratio of PLA/ PLA/nβ-TCP composites after 14 and 21 days of immersion in SBF.
